# Discovery of bacteriorhodopsins in Haloarchaeal species isolated from Indian solar salterns: deciphering the role of the N‐terminal residues in protein folding and functional expression

**DOI:** 10.1111/1751-7915.13359

**Published:** 2019-01-16

**Authors:** Dipesh Kumar Verma, Ishita Baral, Atul Kumar, Senthil E. Prasad, Krishan Gopal Thakur

**Affiliations:** ^1^ Structural Biology Laboratory G. N. Ramachandran Protein Centre Council of Scientific and Industrial Research‐Institute of Microbial Technology (CSIR‐IMTECH) Chandigarh 160036 India; ^2^ Biochemical Engineering Research and Process Development Centre Council of Scientific and Industrial Research‐Institute of Microbial Technology (CSIR‐IMTECH) Chandigarh 160036 India

## Abstract

Interesting optical and photochemical properties make microbial rhodopsin a promising biological material suitable for various applications, but the cost‐prohibitive nature of production has limited its commercialization. The aim of this study was to explore the natural biodiversity of Indian solar salterns to isolate natural bacteriorhodopsin (BR) variants that can be functionally expressed in *Escherichia coli*. In this study, we report the isolation, functional expression and purification of BRs from three pigmented haloarchaea, wsp3 (water sample Pondicherry), wsp5 and K1^T^ isolated from two Indian solar salterns. The results of the 16S rRNA data analysis suggest that wsp3, wsp5 and K1^T^ are novel strains belonging to the genera *Halogeometricum, Haloferax and Haloarcula* respectively. Overall, the results of our study suggest that 17 N‐terminal residues, that were not included in the gene annotation of the close sequence homologues, are essential for functional expression of BRs. The primary sequence, secondary structural content, thermal stability and absorbance spectral properties of these recombinant BRs are similar to those of the previously reported *Haloarcula marismortui* HmBRI. This study demonstrates the cost‐effective, functional expression of BRs isolated from haloarchaeal species using *E. coli* as an expression host and paves the way for feasibility studies for future applications.

## Introduction

Due to the constant depletion of existing fossil fuel reserves and global climate change, there is an urgent need to exploit alternate renewable energy sources to meet ever‐increasing energy demands. One potential solution for harvesting abundant solar energy is a simple, small membrane protein called bacteriorhodopsin. BR was first isolated from a strain of *Halobacterium halobium* and has been extensively studied (Oesterhelt and Stoeckenius, 1971; Lozier *et al*., 1975). BR has several interesting optical and photochemical properties due to its specific molecular structure and specific branched photocycle (bR, K, L, M, N and O). Not all steps in the BR photocycle are photo‐reversible. This vectorial proton transfer property is useful in photoelectric applications. One of the important and last key intermediates of the BR photocycle is the Q state, which is highly stable. BR can remain in the Q state for 7 to 12 years and then returns to its normal bR state upon red photon illumination (Stuart *et al*., 1996, 2002). These unique features coupled with high stability make BR a versatile biological material with numerous potential attractive applications in photovoltaic cells (Hong, 1994; Xu *et al*., 2003; Chellamuthu *et al*., 2016), artificial retina (Chen and Birge, 1993; Cutsuridis and Wennekers, 2009), biosensors (Boucher *et al*., 1996; Lanyi and Luecke, 2001), optical memory storage devices (Birge *et al*., 1990, 1999; Stuart *et al*., 1996, 2002) and optogenetics (Lindvold and Lausen, 2006; Fábián *et al*., 2011). Several attempts to recombinantly express *H. salinarum* BR using *E. coli* as a host have been reported. However, when *H. salinarum* BR is over‐expressed in *E. coli,* it is likely not integrated into the cell membrane, resulting in the formation of inclusion bodies, which are prone to degradation by proteases (Karnik *et al*., 1987). To address this issue, several N‐terminal exogenous tags have been used to help BR integrate into the bacterial inner membrane and increase the stability and yield to some extent. Examples include the use of carrier proteins such as a Mistic (Kahaki *et al*., 2014), MBP (Chen and Gouaux, 1996), β‐lactamase (Karnik *et al*., 1987; Thombre *et al*., 2016) and ompA (Karnik *et al*., 1990). However, the heterologous expression of BR using *E. coli* as an expression host has had limited success in reducing production costs partly due to the need for refolding in the presence of expensive detergents and lipids and/or the removal of recombinant fusion tags using expensive proteases. In a recent report using a chimeric protein strategy and mRNA optimization, ~4 to 9 mg l^−1^ yield of *H. salinarum* point variants was achieved (Bratanov *et al*., 2015).

BRs are widespread and have been reported from diverse microbial sources. This accessibility provides an opportunity to exploit the natural diversity of BRs present in microbes and screen for variants with the desired physicochemical properties and high expression in functional form in *E. coli*. Recently, HmBRI (*Haloarcula marismortui* bacteriorhodopsin‐1), one of the two BRs identified in *Haloarcula marismortui* that shares 52% sequence identity with *H. salinarum* NRC‐1 BR (Fu *et al*., 2010), has been shown to express well in *E. coli* without any fusion tag (Hsu *et al*., 2013), thus overcoming the limitation of poor expression and hope for exploring new BRs for commercial and research applications. Indian solar salterns have not been extensively explored for identifying and characterizing BR‐harbouring haloarchaea. A few studies have reported the identification of BR‐producing haloarchaeal strains from India (Kanekar *et al*., 2015; Thombre *et al*., 2016), but the BRs reported in these studies have not been purified and characterized in detail.

In this study, three pigmented haloarchaeal isolates designated wsp3, wsp4 and wsp5 were isolated from a Pondicherry solar saltern. In addition, one isolate belonging to *Haloarcula sp*. K1^T^, now designated K1^T^, was isolated from the Thamaraikulam Kanyakumari coast of The Bay of Bengal, India. Using PCR‐based screening, we were able to confirm the presence of bacterio‐opsin (*bop*) gene in all four isolates. The genes were cloned in suitable *E. coli* expression vectors. We used several *E. coli* expression hosts and fusion protein strategies but could not achieve functional expression of the BRs. Even attempts to refold BR using published protocols did not yield soluble functional protein. Since all of the BRs share high sequence identity with HmBRI, which is well expressed in *E. coli*, we created several chimeric protein variants. Chimeric proteins in which the 17 amino acid (17 aa) residue N‐terminal region of HmBRI was fused to the sequences of the BRs were soluble and functionally expressed. In addition, we created new protein constructs with extended native sequences at the N‐terminus, which also resulted in soluble protein expression, suggesting errors in the original gene annotations. All BRs reported in this study were well expressed in *E. coli* compared with the native *H. salinarum* BR and were moderately thermostable. The successful expression of the recombinant BRs using *E. coli* reported in this study provides a basis for exploiting these proteins for industrial/commercial applications and for correcting annotation errors in submitted genomes.

## Results

### Screening and identification of BR‐harbouring strains from Indian solar salterns

**Figure 1:**
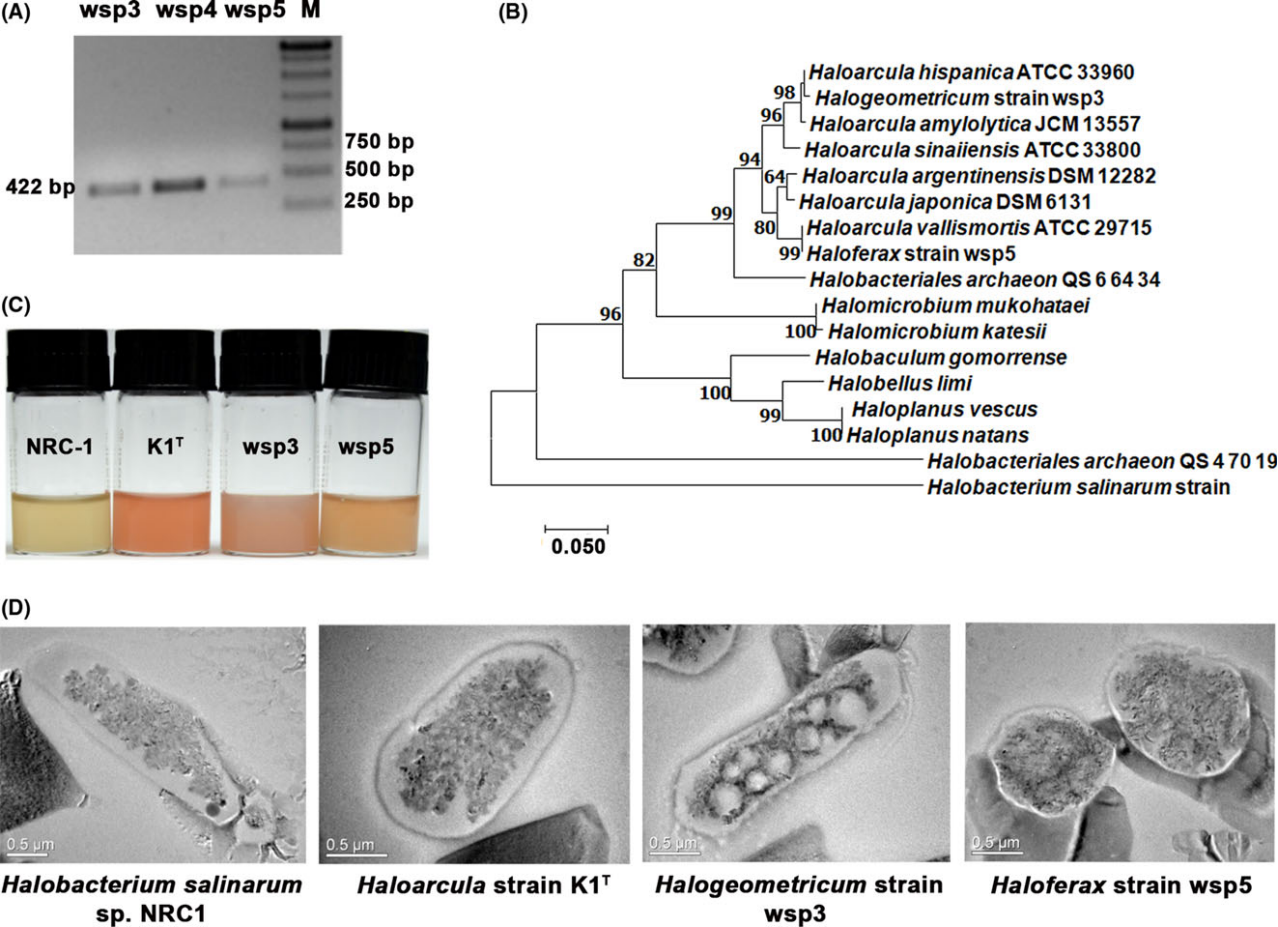
Morphological and taxonomical characterization of the Indian solar saltern haloarchaeal isolates. A. Screening of the isolates with degenerate primers revealed a 410‐bp *bop* positive band upon agarose gel electrophoresis. B. Phylogenetic analysis of the partial *bop* sequences revealed that the wsp3 and wsp5 *bops* have high sequence similarity with the *Haloarcula hispanica* and *Haloarcula argentinensis bops* respectively. C. Wild‐type haloarchaeal cultures of K1^T^, wsp3 and wsp5 appeared more red or pink in colour, possibly due to high carotenoid production compared with *H. salinarum* sp. NRC‐1. D. Transmission electron microscopy images of the isolates suggested pleomorphic morphologies of all three isolates, while *H. salinarum* NRC‐1 has a rod‐shaped morphology.

We isolated four pigmented strains from the Pondicherry and Kanyakumari solar salterns in India. The strains were screened for two properties: the ability to grow at high salt conditions (3–5 M) and the presence of pigments. We assumed that pigmented strains might have a higher probability of harbouring *bop*. Multiple sequence alignment suggested that the nucleotide sequence encoding the N‐termini of the third (N‐α3) and seventh alpha helices (N‐α7) of BR are highly conserved in both *Haloarcula* and *Halobacterium* species. Thus, degenerate primers were designed against these regions (Table S1). The isolated strains were screened for the presence of BR using a PCR‐based screening assay. PCR‐based amplification confirmed the presence of *bop* in all four strains (Fig. 1A). DNA sequencing followed by phylogenetic analysis with 98% boot strap values, performed as described in the ‘Experimental procedures’,showed that the partial *bop* fragments (422 bp) shared maximum sequence similarity (> 98%) with *Haloarcula argentinensis* DSM 12282 (Accession no‐AOLX01000017) and *Haloarcula hispanica* ATCC 33960 (Accession no‐CP00291). The terminal nucleotide sequences of all *Haloarcula* members are highly similar. Therefore, based on the available full‐length *bop* sequences of *Haloarcula hispanica* ATCC 33960 and *Haloarcula argentinensis* DSM 12282 strains (as annotated at National Center for Biotechnology Information (NCBI)), primers were designed to amplify full‐length *bop* (Table S1). Full‐length *bop* sequences (~750 bp) were successfully amplified from all four samples. The wsp3 and wsp4 isolates had identical BR sequences, while K1^T^ and wsp5 BRs shared 99% and 90% sequence identities with the wsp3 BR respectively (Fig. 1B). Therefore, only the wsp3, wsp5 and K1^T^ BRs were further characterized**.**

### Taxonomic classification and morphological characterization of wsp3, wsp5 and K1^T^

The ability to grow under high salt (3 M‐5 M) conditions suggested that the wsp3, wsp5 and K1^T^ strains may belong to the extreme halophilic members of the Halobacteriaceae family. The 16S rRNA sequences of wsp3, wsp5 and K1^T^ (Accession no‐LRHL00000000) have > 99% sequence similarity with *Halogeometricum borinquense* PR3 (Accession no‐NR_102892), *Haloferax volcanii* DS2 (Accession no‐NR_074218.1) and *Haloarcula hispanica* strain Y‐27 (Accession no‐NR_028159) type strains. Only four members of the *Halogeometricum* genus have been reported previously, and only *Halogeometricum rufum* strain CGMCC1.7736 is known to harbour a chromosomal *bop* (Accession no‐FOYT01000001). Similarly, 12 members of the *Haloferax* genus are known, and only the *Haloferax mucosum* ATCC BAA‐1512 strain has a gene annotated as *bop* (Accession no‐WP_008320464.1). Therefore, both wsp3 and wsp5 are the second *bop‐*harbouring members in their respective genera. We also performed whole‐genome sequence analysis of K1^T^, which suggested that it harbours a chromosomal *bop* with an operon structure similar to that observed in other species in the genus *Haloarcula*. In addition, the appearance of pink‐red colour upon culturing indicated that the wsp3, wsp5 and K1^T^ strains are high carotenoid producers compared with *Halobacterium salinarum* NRC‐1 (Fig. 1C) (Grant *et al*., 2001; Mandelli *et al*., 2012). *Halobacterium salinarum* is a rod‐shaped haloarchaeon, while transmission electron microscopy (TEM) images suggested that wsp3, wsp5 and K1^T^ have highly pleomorphic morphologies (Figs 1D and S1). The TEM images also suggested that wsp3 is highly vacuolated compared with other Haloarchaea (Figs 1D and S1).

### Evaluating a fusion tag‐based strategy for the recombinant and functional expression of BR

Cultures of BR‐expressing transformants were supplemented with trans‐retinal to facilitate BR folding and maturation (Braiman *et al*., 1987). However, we did not observe any colour after harvesting the cells. Purification of BR was attempted using Ni‐NTA chromatography, as described in the Experimental Procedures section, but no protein band corresponding to BR was observed after SDS‐PAGE, perhaps due to the instability and protease sensitivity of recombinant BR (Cunningham and Deber, 2007). The Mistic tag has been shown to improve the expression of BR (Nekrasova *et al*., 2010; Kahaki *et al*., 2014). Four Mistic variants, M110, M2, M3 and M4, have been reported in the literature (Roosild *et al*., 2006; Dvir and Choe, 2009). All of the target BR genes were cloned in fusion with a recently identified Mistic tag (M4) isolated from *Bacillus atrophaeus,* and the fusion proteins were purified as described in the Experimental Procedures section. The M4‐BR fusion proteins expressed well, and we successfully purified the target proteins (Fig. S2). However, the M4‐BR fusion proteins were not stable, as evidenced by the degradation products observed upon SDS‐PAGE (Fig. S2). We followed the published protocol to refold BR (Kahaki *et al*., 2014). The only variation was that the on‐column cleavage of the Mistic tag was performed using the Tobacco Etch Virus (Marque *et al*., 1984) protease. The cleaved BR was refolded in the presence of phospholipids and retinal, but we did not observe any peak at 400–650 nm in UV–vis spectroscopy, suggesting that the protein did not adopt its native conformation and therefore failed to bind retinal.

### Effective expression of chimeric BRs in functional form in E. coli

**Figure 2:**
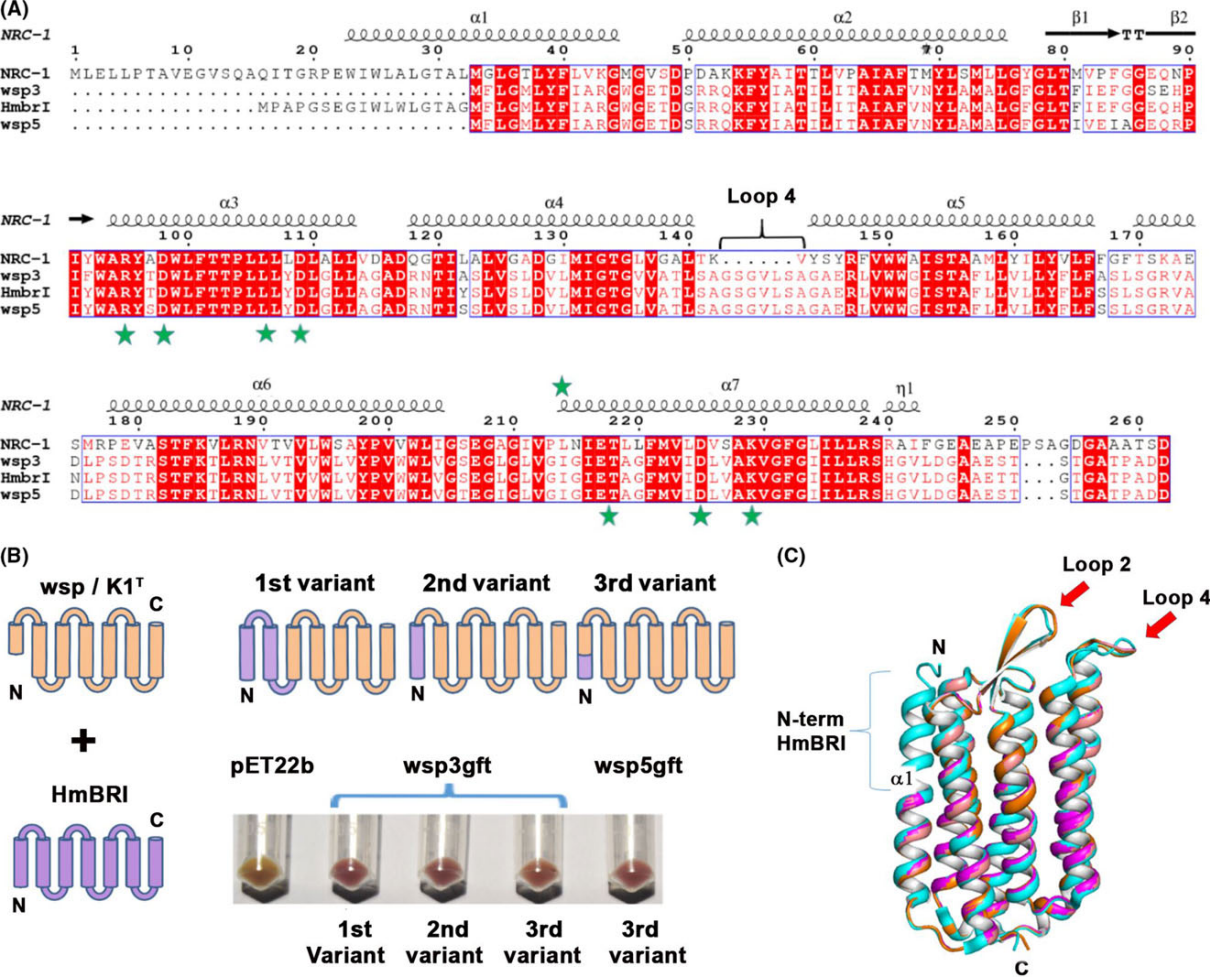
Probing the role of the N‐terminal residues in the functional expression of BR in the heterologous host. A. Multiple sequence alignment of *H. salinarum* BR with HmBRI, wsp3, wsp5 and K1^T^ BRs. Wsp5 BR has maximum identity with HmBRI ^D94N^, and only one amino acid differs between the wsp3 and K1^T^ BRs. Conserved residues involved in proton pumping are highlighted by the green‐coloured stars. B. The protein engineering strategy that was employed to create the chimeric BR variants. All three variants resulted in retinal‐bound coloured protein expression. C. Structural superposition of the models of the wsp3 (orange), wsp5 (pink) and K1^T^ (purple) proteins on the crystal structure of HmBRI (cyan) (PDB ID 4PXK) suggested that the grafted N‐terminal region adopts α‐helical conformation.

Multiple sequence alignment of the wsp BRs isolated in this study with HmBRI from *H. marismortui* revealed the presence of an additional 17 aa residues at the N‐terminus in the latter (Fig. 2A). Since HmBRI is the only known BR that is highly functionally expressed in *E. coli* without any fusion tag, we hypothesized that the presence of the N‐terminal 17 aa residues is crucial for protein expression and folding. Thus, we created chimeric BRs in which we grafted three different stretches of the N‐terminal region of HmBRI on the target BR (wsp3, wsp5 and K1^T^). The three regions consist of α1‐loop1‐α2‐loop2 (residue range 1–78), α1 (residue range 1–29 amino acid) or the N‐terminal region of α1 (residue range 1–17) (Fig. 2B and C).

**Figure 3:**
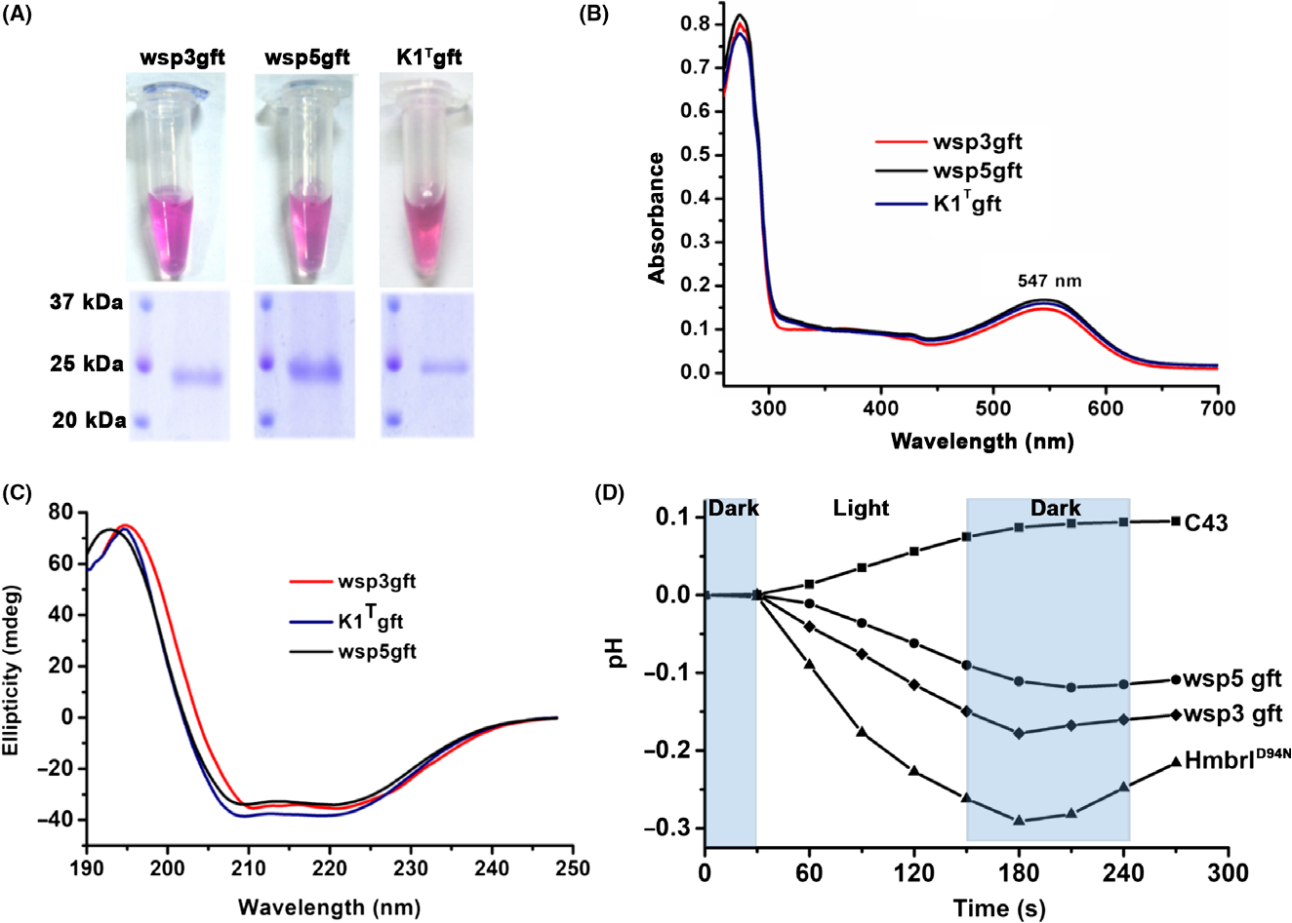
Chimeric BRs expressed in functional form in *E. coli*. A. The purified N‐terminal grafted BR constructs (wsp3gft, wsp5gft and K1^T^gft) showed red‐pink coloured proteins on SDS‐PAGE. B. Spectral measurement data showing the 547‐nm absorbance of all grafted BRs. C. Circular dichroism (CD) data confirmed the folding of the α‐helical secondary structure of the N‐term grafted BRs. D. *E. coli* cells expressing grafted BRs were incubated with continuous light and dark cycles to measure proton pumping activity. C43 represents *E. coli* BL2‐C43 cells used as a negative control in the experiments.

The clones were confirmed by DNA sequencing and transformed in *E. coli* BL21 (DE3) C43‐pRARE cells (*E. coli* BL21 (DE3) C43 cells harbouring the pRARE plasmid) to assess expression. One reason for poor expression of heterologous proteins in an expression host is the presence of rare codons in the gene. Bioinformatics‐based analysis revealed the presence of rare codons in the *bops*. Therefore, we used *E. coli* BL21 (DE3) C43 competent cells harbouring the pRARE plasmid to aid the expression of the BRs. The pRARE plasmid (isolated from Rosetta DE3 cells, Novagen) encodes proL‐tRNA, leuW‐tRNA, argW‐tRNA, thrT‐tRNA, glyT‐tRNA, argU‐tRNA and ilex‐tRNA, all rare codons in *E. coli* that help to enhance the expression of proteins encoded by genes with rare codons (Ikemura, 1981). Retinal was added post‐induction, and upon harvesting, we observed coloured cell pellets, suggesting the functional expression of the chimeric BRs (Fig. 3A).

The chimeric BRs were purified using Ni‐NTA‐based affinity chromatography. Optimal n‐dodecyl‐β‐d‐maltoside (DDM) detergent concentrations were maintained throughout the purification steps. Protein bands of the expected molecular weights were observed on SDS‐PAGE (Fig. 3A). We successfully purified 2–4 mg l^−1^ protein, which is comparable to other known recombinant BR expression systems (Fu *et al*., 2010). The identity of the proteins was further confirmed using peptide mass fingerprinting (Fig. S3). Interestingly, all the chimeric BR variants were expressed in the functional form, suggesting that the fusion of the N‐terminal 17 residues from HmBRI was sufficient for functional BR expression.

It has been reported that the N‐terminal region of *H. salinarum* BR is also important for protein folding and membrane integration (Mogi *et al*., 1989). The expression of functional chimeric BRs also suggests that the wsp3, wsp5 and K1^T^ BRs, which have a truncated α1, may not integrate well in the membrane and are possibly degraded by proteases, which could explain the failure to observe protein expression from constructs designed based on the available gene annotation data. We also used the PHyRe2 webserver (Kelley *et al*., 2015) to build structural models of the wsp3, wsp5 and K1^T^ BRs. These modelled structures were superimposed on the crystal structure of HmBRI^D94N^ BR (PDB ID 4PXK) (Hsu *et al*., 2013). These data demonstrated that grafting the N‐terminal residues aid the formation of the first helix of the protein, thus helping to stabilize the structure and possibly membrane integration.

UV–vis absorbance spectrum analysis revealed an absorbance maxima of ~547 nm (Fig. 3B), whereas HmBRI reportedly has an absorbance maxima at 552 nm. The shift of ~5 nm could be due to the sequence variations close to the retinal binding site. In addition, circular dichroism analyses also confirmed that the purified proteins had α‐helical secondary structural contents and were folded (Fig. 3C).

### Wsp3gft, wsp5gft and HmbrI^D94N^ BRs exhibit light‐driven proton pumping activity

Haloarcheal BRs have light‐driven proton pumping activity. Recombinant proteorhodopsin (PR) expressed in *E. coli* can pump protons across the membrane as indicated by a decrease in pH (Wang *et al*., 2003). We therefore used this strategy to test the light‐driven proton pumping activity of wsp3gft and wsp5gft. We also included a codon‐optimized version of the *H. marismortui* HmBRI‐D94N BR (HmBRI^D94N^) mutant for comparative analysis with the wsp BRs. HmbrI^D94N^ was selected because it is highly expressed compared with wild‐type HmBRI (Hsu *et al*., 2013). Briefly, the BR‐producing *E. coli* cells were pelleted, followed by washing with a non‐buffered solution. The cells were finally resuspended in non‐buffered solution, and the change in pH was monitored using a pH metre. Under dark conditions, the pH was stable, but when we flashed white light, there was a significant decrease in the pH compared with the control cells that did not express recombinant BR. After switching off the light, an increase in pH was observed, which may be due to reversed proton flow, possibly through the ATPase complex (Fig. 3D). This assay was performed using a similar number of *E. coli* cells expressing the respective BRs. We consistently observed a larger decrease in pH in the HmBRI^D94N^‐expressing cells. This difference may be due to the higher expression level of the D94N mutant of HmBRI than of the other BRs in our protein expression and purification experiments (data not shown). These data demonstrate that the BRs isolated from the Indian solar salterns were expressed well in functional form in *E. coli*.

### Errors in bop gene annotation in the genus Haloarcula

The genome of K1^T^ was initially annotated using the reference strains *Haloarcula hispanica* ATCC 33960 and *Haloarcula argentinensis* DSM 12282. Interestingly, all of the BRs annotated in these genomes are shorter than HmBRI. Our results presented above led us to re‐examine the genome sequence to identify potential annotation errors or any differences in the N‐terminal regions of BRs. The 17 aa encoded by the upstream region of K1^T^ share 88% sequence identity with HmBRI. Therefore, we suggest that these genes were misannotated and that the correct genes should include additional N‐terminal sequences as well.

### Expression of the K1^T^ BR from the alternate translation start site yields functional expression

**Figure 4:**
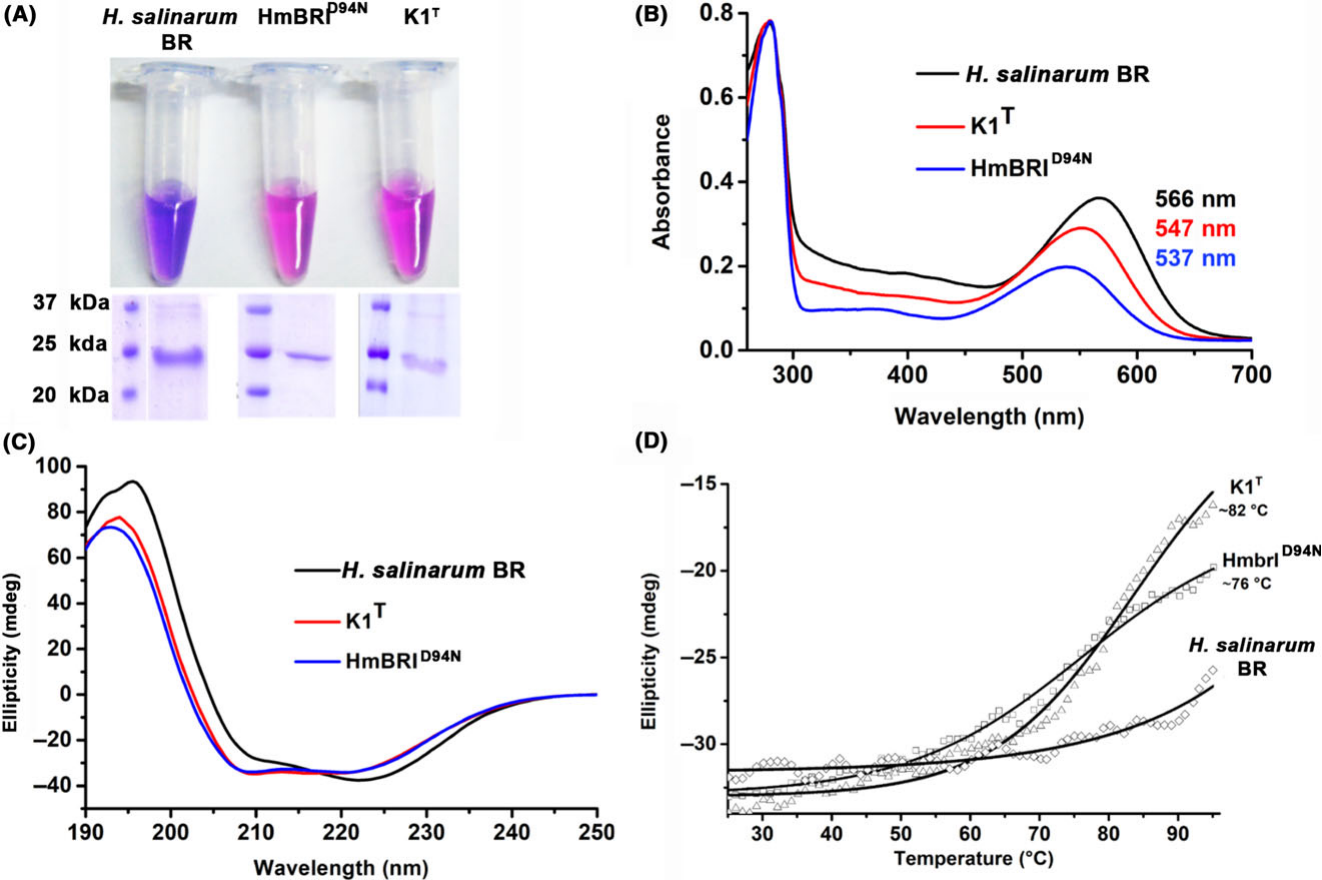
Structural and thermal stability comparison of the native *H. salinarum* BR and the recombinant BRs purified in this study. A. The purified native *H. salinarum* BR and recombinant BRs were resolved by SDS‐PAGE. B. UV‐Vis absorbance spectra of K1^T^, HmBRI ^D94N^ and *H. salinarum* BRs. C. CD spectral analysis revealed that the K1^T^ and HmBR1^D94N^ BRs adopted similar secondary structure profiles. However, *H. salinarum* BR showed a visually distinct profile. All three proteins had > 83% α‐helical secondary structural contents. D. Thermal melting profiles of the recombinant BRs and native *H. salinarum* BR.

Using the whole‐genome sequence information of K1^T^, we designed a new construct that included the 17 residues upstream of *bop*. This construct was cloned in the vector pET22b to yield a recombinant protein with a C‐terminal 6x His tag. This protein construct was also expressed in functional form with absorbance maxima comparable to those of the chimeric BRs (Fig. 4A). Circular dichroism analysis of the full‐length K1^T^ BR suggested that it was folded (Fig. 4C). Interestingly, circular dichroism (CD) analysis of *H. salinarum* BR, K1^T^ and HmBRI using the K2D3 web server (http://cbdm-01.zdv.uni-mainz.de/~andrade/k2d3) suggested that both K1^T^ and HmBRI contain approximately 84% α‐helical secondary structure content, comparable to ~83% in *H. salinarum* BR. Although the secondary structure contents are comparable, the CD profile of *H. salinarum* BR is visually distinct from those of K1^T^ and HmBRI (Fig. 4C). Detergent solubilization probably removed most of the native archaeal lipids, but some percentage were still bound, and this change in the lipid–protein ratio probably altered the CD profile of *H. salinarum* BR (Mao and Wallace, 1984). CD profile of *H. salinarum* BR is similar to the low lipid‐BR protein profile. On the other hand, the CD profile of the recombinant *H. salinarum* BR expressed and purified from *E. coli* has greater similarity with our HmBRI, wsp3 and wsp5 recombinant BRs (Nekrasova *et al*., 2010).

### Thermal stability of the recombinant BRs

For some industrial or commercial applications of BR, it is desirable to have proteins with high to moderate thermal stability. As reported earlier, *H. salinarum* BR has high thermal stability (Marque *et al*., 1984; Brouillette *et al*., 1989) and thus is suitable for many commercial applications. We solubilized both native and recombinant BR in detergent and performed CD spectroscopy‐based thermal denaturation experiments to examine the thermal stability of the recombinant BRs (HmBRI^D94N^ and K1^T^) compared with *H. salinarum* BR. The recombinant BRs had a *T*_m_ of approximately 78°C, while *H. salinarum* BR had high thermal stability, as its secondary structure content was only partially lost even at 95°C (Fig. 4D). When we incubated the BRs at 75°C for ten minutes, both HmBRI^D94N^ and K1^T^ lost the bound retinal, while *H. salinarum* BR retained its colour (Fig. S4). These data also suggest that HmBRI^D94N^ and K1^T^ have moderate thermal stability. Although both of these recombinant proteins are less stable than *H. salinarum* BR, they can be explored for applications where moderate thermal stability will suffice.

### Expression of full‐length bop constructs

**Figure 5:**
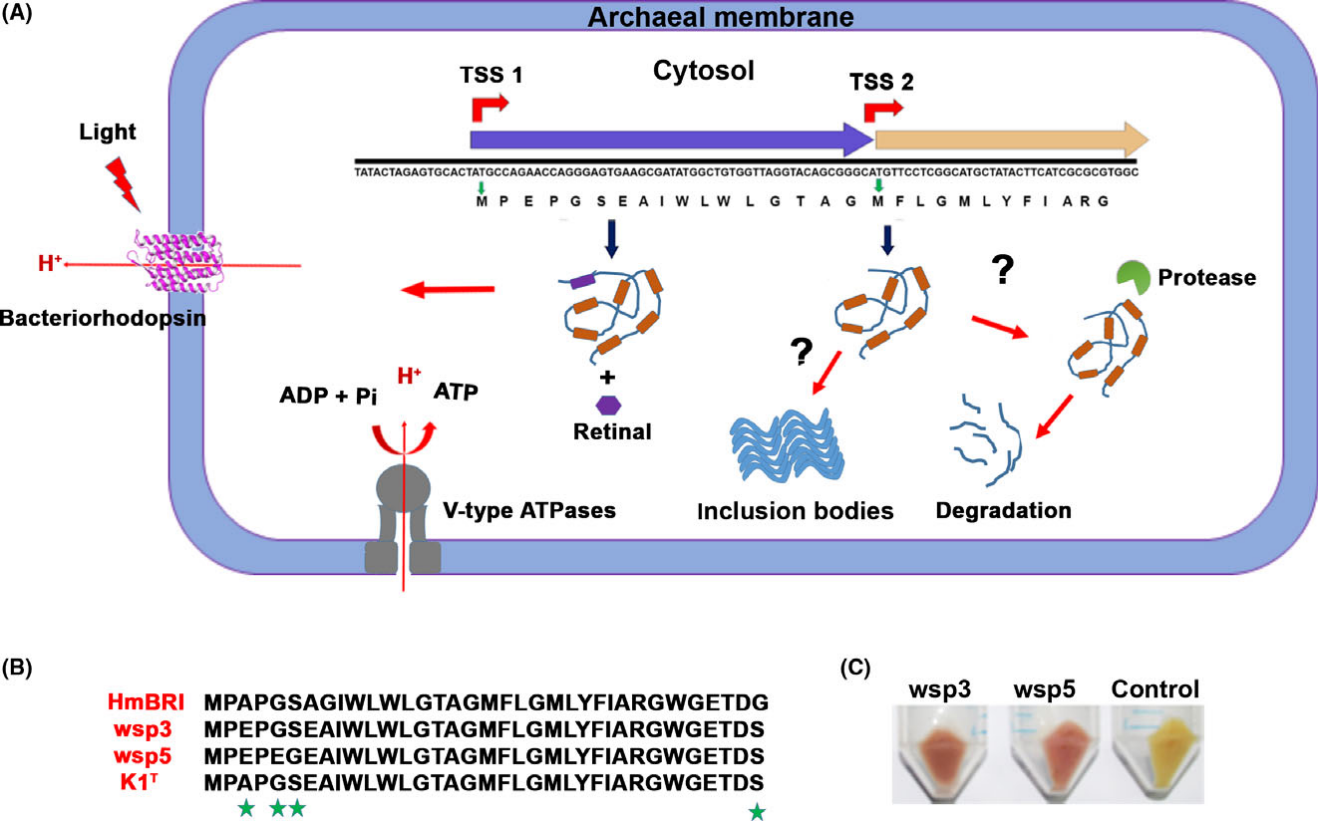
Gene annotation errors in *bops* belonging to the genus *Haloarcula*. A. *Haloarcula* *bops* have two alternate translation start sites. Protein translation from start site 2 results in a truncated protein that forms inclusion bodies or is probably degraded by cellular proteases. By contrast, translation of the BR product from start site 1 results in functional expression and successful integration in the cell membrane. B. N‐terminal sequence comparison suggested that the proteins are highly similar with few amino acid variations. C. A red‐coloured pellet was observed after the expression of the constructs designed using start site 1 for the wsp3 and wsp5 BRs, suggesting functional expression of the BRs.

Multiple sequence alignment of wsp3, wsp5, K1^T^ and HmBRI^D94N^ suggested that all of the BRs have 97–98% sequence identity, with the exception of a few residues in the loop region second. Therefore, full‐length wsp3 and wsp5 were also amplified using a set of primers similar to those used for K1^T^ and cloned in the pNIC‐Bsa4 LIC vector with a C‐terminal 6× His tag. The full‐length constructs were also expressed effectively and yielded coloured retinal‐bound recombinant BRs (Fig. 5C).

### Comparative analysis of the bop operons in haloarchaea

We analysed the annotated genomes available for the *Haloarcula, Halogeometricum* and *Haloferax* genera to understand the arrangement of the genes involved in BR synthesis and regulation. The *bop* operons for *H. salinarum* and other species are shown in Fig. S5. In *Haloarcula sp.,* the *bop* gene is positioned between the B and D subunits of V‐type ATPases (Fig. S5). Previous RT‐PCR‐based analyses have indicated that *bop* is expressed independent of environmental illumination in *H. marismortui* (Fu *et al*., 2010), while the level of *bop* expression in *H. salinarum* is highly regulated in response to various factors, such as light, oxygen and pH (Oesterhelt and Stoeckenius, 1973). The physiological role of *bop* in *H. marismortui* is not well understood. However, Fu *et al*. have reported that the presence of HmBRI and HmBRII dual rhodopsin system, where HmBRI is functional at pH > 5.0 and HmBRII is functional at pH > 4.0, helps *H. marismortui* to survive at low pH conditions in high salinity environments like that present in the Dead Sea (pH ~ 5.0) (Fu *et al*., 2013).

Whole‐genome data analyses are available for type strains of *H. borinquense* and *H. volcanii*, and surprisingly, no *bop* is present in these genomes (Fig. S6). Moreover, the V‐type ATPase operon is present, and there is no gene insertion in this operon. In *Halogeometricum rufum* strain CGMCC1.7736*,* the V‐type ATPases operon contains two extra genes encoding PAS domain‐containing protein (Accession no‐ WP_089804275) and lycopene cyclase (Accession no‐ WP_089804279) along with the *bop* gene. These data suggest that *bop* was probably inserted in *H. marismortui* and other *Haloarcula sp*., possibly due to horizontal gene transfer.

## Discussion

Microbes living in extreme environmental conditions have evolved mechanisms and possess genes that aid their survival and adaptation. Although most of the genes are encoded in the chromosome, the acquisition of new genetic content by horizontal gene transfer can aid in microbial evolution and adaptation. In this study, water samples were collected from two Indian solar salterns separated by a distance of approximately 600 km. The major objective of this study was to identify BR‐harbouring haloarchaeal strains to test natural BR variants for high recombinant expression using *E. coli* as an expression host. We successfully isolated three *bop*‐harbouring strains from two Indian solar salterns.

BR helps haloarchaea produce ATP under nutrient‐limiting conditions based on its light‐driven proton pumping activity (Danon and Stoeckenius, 1974; Bogomolni *et al*., 1976; Bogomolni, 1977). The haloarchaeon *H. salinarum* has been studied extensively for BR production and its applications. Previously reported recombinant methods for BR purification to have limitations, such as the need for protease digestion to remove the purification tags, refolding in the presence of expensive phospholipids (Opekarova and Tanner, 2003; Kahaki *et al*., 2014), multistep purifications and susceptibility of the recombinant proteins to degradation. Bratanov *et al*. (2015) reported a method to improve recombinant BR expression and help reduce its expression cost. We tested several detergents, and we could successfully solubilize wsp BRs using DDM.

After facing initial challenges due to annotation errors in the genomes, we were able to achieve functional expression after including approximately 17 aa in the N‐terminal region of the BRs. Structural analysis revealed that the N‐terminal 17 aa is part of the N‐terminal helix and therefore may be required for protein folding. The deletion of this region results in protein degradation or may affect the membrane integration of the leaderless BRs.

Our study and previous reports demonstrate that industry‐friendly and cost‐effective recombinant expression of functional BR is possible using fast‐growing *E. coli* hosts. This strategy employs *in situ* retinal binding and single‐step purification using Ni‐NTA affinity chromatography and has the potential to significantly reduce the production cost of BR variants. This method also alleviates the need for protein refolding in the presence of expensive detergents or membrane mimetics or removing purification tags using expensive proteases. However, the photochemical properties of these BR variants need to be thoroughly investigated for their suitability in commercial applications. In addition, if the need arises, these proteins can be easily subjected to directed evolution to improve or engineer the desired properties.

Our study also suggests that the *bop* gene has two possible translation start sites (TSS‐1 and TSS‐2) for BR and that translation from TSS‐1 leads to functional BR protein expression. By contrast, the expression constructs generated from the TSS‐2 results in a truncated non‐functional protein. The *bop* gene sequences available in the NCBI protein repository database suggest that many *bop* genes are annotated from TSS‐2. Some of these errors have been corrected recently, but several strains, such as *Haloarcula hispanica* ATCC 33960 (GenBank: AEM56052.1), *Haloarcula amylolytica* JCM 13557 (GenBank: EMA19462.1), *Halobacterium jilantaiense, Haloarcula rubripromontorii* strain SL3 (GenBank: KOX94702.1), *Haloarcula sinaiiensis* ATCC 33800 (GenBank: EMA12126.1), *Haloarcula japonica* DSM 6131(GenBank: EMA28897.1), *Haloarcula vallismortis* ATCC 29715 (GenBank: EMA00745.1), *Halorubrum kocurii* JCM 14978 (EMA62282.1) and *Haloarcula argentinensis DSM 12282* (GenBank: EMA22434.1), are still annotated from TSS‐2. In addition to *Halobacterium* and *Haloarcula,* many other Haloarchaea harbouring *bop* have similar annotation errors that need to be corrected.

In conclusion, this study introduces new natural BR variants that are expressed well in *E. coli* and can be further explored for various industrial and research applications. This study also highlights the importance of studying natural diversity to isolate proteins with desirable properties and illustrates that errors in genome annotation can result in the improper design of expression constructs, leading to the expression of non‐functional proteins.

## Experimental procedures

### Solar saltern sample collection and screening for extreme halophiles

Pigmented haloarchaeal isolates were obtained from solar saltern samples collected from the Marakkanam solar salterns (12°11′13.0272″N and 79°55′40.4220″E) and the Pondicherry and Thamaraikulam solar salterns (8°07′07.30″N and 77°29′13.76″E) in Kanyakumari, India, by the membrane filtration technique as suggested by Montalvo‐Rodriguez *et al*. (1998) and Elevi *et al*. (2004). Saline water samples (250 ml) were filtered through 0.45‐micron membrane filters using vacuum filtration techniques. The membrane filters containing pink‐red patches were transferred onto sterile HB medium consisting of 250 g l^−1^ NaCl, 20 g l^−1^ MgSO4. 7H_2_0, 3 g l^−1^ trisodium citrate, 2 g l^−1^ KCl and 10 g l^−1^ Oxoid peptone. The pH was adjusted to approximately 7.2–7.4 using 10 N NaOH.

### Isolation of extreme halophiles

After 7–8 days of incubation, colonies with a red‐pink colour appeared on the membrane filter. The pieces of membrane filters containing pink‐red pigmented colonies were transferred to 50 ml of HB medium in a 500‐ml flask for incubation at 37°C and 200 rpm. The grown cultures (after 4–5 days of incubation) were streaked onto HB agar plates. This subculturing process was repeated three times to isolate pure single colonies. After 7–8 days of incubation at 37°C, pink and red colonies were observed on the agar Petri plates. Four pigmented strains designated wsp3, wsp4, wsp5 and K1^T^ were isolated using this protocol.

### DNA extraction and 16S rRNA gene sequencing

Genomic DNA of the wsp3 and wsp5 strains was isolated using a Zymogen DNA isolation kit (Cat No. D6105, ZYMO RESEARCH), and the 16S rRNA gene sequences were amplified by PCR. The amplified products were run on 1.0% agarose gels and further extracted using a PCR clean‐up kit (Thermo Scientific, Cat No. K0701). The taxonomic identification and percentage similarity of the amplified 16S rRNA gene sequences were calculated using the ‘Identity’ option of the EzTaxon e‐server (http://www.eztaxon.org). The evolutionary history was inferred using the neighbour‐joining method (Saitou and Nei, 1987). The optimal tree with the sum of branch length = 1.21597215 is shown. The percentage of replicate trees in which the associated taxa clustered together in the bootstrap test (1500 replicates) are shown next to the branches (Felsenstein, 1985). The tree is drawn to scale, with branch lengths in the same units as those of the evolutionary distances used to infer the phylogenetic tree. The evolutionary distances were computed using the Poisson correction method and are in the units of the number of amino acid substitutions per site. The analysis involved 18 amino acid sequences. All positions containing gaps and missing data were eliminated. There were a total of 126 positions in the final data set. Evolutionary analyses were conducted in MEGA7 (Kumar *et al*., 2016).

### Cloning, expression and purification of BRs

The amplified *bops* were cloned in pNIC28‐Bsa4 and pET22b between the *Nhe*‐*Xho*I restriction sites for expression as C‐terminal 6× His‐tagged proteins or the pET28a vector (modified) between the *NdeI‐Xho*I sites for expression as a Mistic fusion protein. Positive clones were transformed into *E. coli* C43‐Rosetta BL21 (DE3) cells for protein expression. Protein expression was induced at A_600_ ~ 0.6 OD with 0.5 mM IPTG. The culture was supplemented with 5–10 μM retinal to enhance BR maturation and functional expression. Trans‐retinal was purchased from Sigma‐Aldrich, (St. Louis, MO, USA) (R2500‐1G), and stocks were prepared in 100% ethanol. The culture was incubated at 37°C and 200 rpm for 5 h in an incubator shaker. The cells were harvested at 9000 × *g* for 10 min and resuspended in lysis buffer A (50 mM Tris and 150 mM NaCl, pH 8.0). The cells were lysed by sonication, and the insoluble membrane fraction was obtained by centrifuging the lysate at high speed at 18 000 × *g* for 30 min. The soluble fraction was discarded, and the pellet was resuspended in lysis buffer B (50 mM Tris and 150 mM NaCl pH 8 with 0.2% DDM). For proteins expressed with the Mistic tag, 0.2% SDS detergent was used instead of DDM. The resuspended insoluble fraction was incubated overnight with gentle shaking. DDM and SDS were used to facilitate BR extraction from the insoluble lipid bilayer, thus solubilizing the protein. The soluble fraction of the protein was mixed with Ni‐NTA resin for binding, and the proteins were eluted using elution buffer E (50 mM Tris and 150 mM NaCl, pH 8.0 with 0.02% DDM or 0.02% SDS in the case of the Mistic tag with 500 mM imidazole).

### BR isolation from wild‐type ET001

Native BR was isolated from the *Halobacterium salinarum* ET001 strain, which was a gift of Professor Marc T Facciotti. Purple membrane was isolated from *Halobacterium salinarum* ET001 cells as described by (Oesterhelt and Stoeckenius, 1973). Briefly, the purple membrane was extracted after sucrose density separation. The isolated purple membrane was collected and dialysed against double‐distilled water using 10‐kDa dialysis membranes at 4°C to remove the sucrose. The final purple membrane pellet was suspended in 20 mM phosphate buffer, pH 7.2 and stored at −20°C for further use.

### Grafting of the wsp3, wsp5 and K1^T^ fragments with HmBRI bop to improve their expression and stability

To improve protein expression, different gene fragments of HmBRI were grafted onto the K1^T^ and wsp *bops* using overlapping primers (Table S1). The grafting was conducted in two steps of amplification. First, the wsp and K1^T^ *bop*s were amplified using forward overlapping primers and gene‐specific reverse primers. The amplified fragments possessed an overlapping region with HmBRI *bop* and were used to amplify the full‐length grafted genes using HmBRI‐specific forward primers. The amplified grafted products were further digested and cloned in pET22b for expression with a C‐terminal His tag.

### BR spectral analysis

The purified proteins were dialysed against buffer B (50 mM Tris, 150 mM NaCl, pH 8, with 0.2% DDM) to obtain a concentration of 10 μM and further subjected to visible range spectral scanning from 200 to 700 nm on a CECIL CE7500 spectrophotometer.

### Circular dichroism analysis of secondary structure and thermal stability

The secondary structure analysis and thermal experiments were performed using a JASCO J‐815 CD spectrometer at a concentration of 10 μm in a 1‐mm path‐length quartz cuvette. The CD data were recorded at 20°C in the far UV range of 190–250 nm at a data pitch of 0.5 nm, scanning speed of 50 nm min^−1^ and bandwidth of 1 nm. Thermal melt experiments were performed starting from a temperature of 25°C and increasing to 95°C with a ramp rate of 1°C min^−1^. The spectra were recorded every 5°C with a scanning speed of 100 nm min^−1^.

### Light‐driven proton pumping assay

The light‐driven proton pumping assay was performed as described by Wang *et al*. (2003). Rhodopsin‐expressing *E. coli* cells were harvested at 1575 × *g* at 4°C, washed twice and resuspended in a non‐buffered solution (10 mM NaCl, 10 mM MgSO_4_ and 100 mM CaCl_2_). The OD A_600_ was adjusted to 2.0 in the dark, and the proton pumping experiment was started by illuminating the cells with a high‐intensity white light source for 120 s. The real‐time rate of the change in pH (ion transport activity) was monitored using a Mettler Toledo pH metre. Similarly, the back flow of protons was monitored based on the increase in pH upon post‐incubation of the culture in the dark.

## Conflict of interest

The authors declare that there is no conflict of interest.

## Supporting information


**Fig. S1.** Morphological variations observed in the haloarchaeal isolates.
**Fig. S2.** Schematic presentation of the Mistic‐BR fusion constructs designed and used in the study.
**Fig. S3.** In‐gel digestion and ms/ms ion search for K1^T^ BR.
**Fig. S4**. Heat denaturation of the wild‐type *H. salinarum* BR and K1^T^ *bop*.
**Fig. S5.** Operon analysis of *bop*.
**Fig. S6**. Operon schematics of V‐type ATPases in (A) *H. borinquense* (B) *H. marismortui* (C) *H. rufum,* showing the *bop* position in between the B and D subunits of the V‐type ATPases.
**Table S1.** Forward and reverse primers for 16S rRNA amplification, degenerative primers, full‐length *bop* amplification and gene grafting.

## References

[mbt213359-bib-0001] Birge, R.R. , Fleitz, P.A. , Gross, R.B. , Izgi, J.C. , Lawrence, A.F. , Stuart, J.A. and Tallent, J.R. (1990) Spatial light modulators and optical associative memories based on bacteriorhodopsin In Proceedings of the Twelfth Annual International Conference of the IEEE Engineering in Medicine and Biology Society. Philadelphia, PA: IEEE, pp. 1788–1789.

[mbt213359-bib-0002] Birge, R.R. , Gillespie, N.B. , Izaguirre, E.W. , Kusnetzow, A. , Lawrence, A.F. , Singh, D. , *et al* (1999) Biomolecular electronics: protein‐based associative processors and volumetric memories. J Phys Chem B 103: 10746–10766.

[mbt213359-bib-0003] Bogomolni, R.A. (1977) Light energy conservation processes in *Halobacterium halobium* cells. Fed Proc 36: 1833–1839. 15879

[mbt213359-bib-0004] Bogomolni, R.A. , Baker, R.A. , Lozier, R.H. , and Stoeckenius, W. (1976) Light‐driven proton translocations in *Halobacterium halobium* . Biochem Biophys Acta 440: 68–88. 7322 10.1016/0005-2728(76)90114-6

[mbt213359-bib-0005] Boucher, F. , Taneva, S.G. , Elouatik, S. , Dery, M. , Messaoudi, S. , Harvey‐Girard, E. , and Beaudoin, N. (1996) Reversible inhibition of proton release activity and the anesthetic‐induced acid‐base equilibrium between the 480 and 570 nm forms of bacteriorhodopsin. Biophys J 70: 948–961. 8789112 10.1016/S0006-3495(96)79638-8 PMC1224995

[mbt213359-bib-0006] Braiman, M.S. , Stern, L.J. , Chao, B.H. , and Khorana, H.G. (1987) Structure‐function studies on bacteriorhodopsin. IV. Purification and renaturation of bacterio‐opsin polypeptide expressed in *Escherichia coli* . J Biol Chem 262: 9271–9276. 3298254

[mbt213359-bib-0007] Bratanov, D. , Balandin, T. , Round, E. , Shevchenko, V. , Gushchin, I. , Polovinkin, V. , *et al* (2015) An approach to heterologous expression of membrane proteins. The Case of Bacteriorhodopsin. PLoS ONE 10: e0128390. 26046789 10.1371/journal.pone.0128390 PMC4457421

[mbt213359-bib-0008] Brouillette, C.G. , McMichens, R.B. , Stern, L.J. , and Khorana, H.G. (1989) Structure and thermal stability of monomeric bacteriorhodopsin in mixed phospholipid/detergent micelles. Proteins 5: 38–46. 2748571 10.1002/prot.340050106

[mbt213359-bib-0009] Chellamuthu, J. , Nagaraj, P. , Chidambaram, S.G. , Sambandam, A. , and Muthupandian, A. (2016) Enhanced photocurrent generation in bacteriorhodopsin based bio‐sensitized solar cells using gel electrolyte. J Photochem Photobiol B. Biology 162: 208–212. 27380296 10.1016/j.jphotobiol.2016.06.044

[mbt213359-bib-0010] Chen, Z. , and Birge, R.R. (1993) Protein‐based artificial retinas. Trends Biotechnol 11: 292–300. 7763952 10.1016/0167-7799(93)90017-4

[mbt213359-bib-0011] Chen, G.Q. , and Gouaux, J.E. (1996) Overexpression of bacterio‐opsin in Escherichia coli as a water‐soluble fusion to maltose binding protein: efficient regeneration of the fusion protein and selective cleavage with trypsin. Protein Sci 5: 456–467. 8868482 10.1002/pro.5560050307 PMC2143362

[mbt213359-bib-0012] Cunningham, F. , and Deber, C.M. (2007) Optimizing synthesis and expression of transmembrane peptides and proteins. Methods 41: 370–380. 17367709 10.1016/j.ymeth.2006.07.003

[mbt213359-bib-0013] Cutsuridis, V. , and Wennekers, T. (2009) Hippocampus, microcircuits and associative memory. Neural Networks 22: 1120–1128. 19647982 10.1016/j.neunet.2009.07.009

[mbt213359-bib-0014] Danon, A. , and Stoeckenius, W. (1974) Photophosphorylation in *Halobacterium halobium* . Proc Natl Acad Sci USA 71: 1234–1238. 4524635 10.1073/pnas.71.4.1234 PMC388199

[mbt213359-bib-0015] Dvir, H. , and Choe, S. (2009) Bacterial expression of a eukaryotic membrane protein in fusion to various Mistic orthologs. Protein Expr Purif 68: 28–33. 19524676 10.1016/j.pep.2009.06.007 PMC2728152

[mbt213359-bib-0016] Elevi, R. , Assa, P. , Birbir, M. , Ogan, A. , and Oren, A. (2004) Characterization of extremely halophilic Archaea isolated from the Ayvalik Saltern, Turkey. World J Microbiol Biotechnol 20: 719–725.

[mbt213359-bib-0017] Fábián, L. , Heiner, Z. , Mero, M. , Kiss, M. , Wolff, E.K. , Ormos, P. , *et al* (2011) Protein‐based ultrafast photonic switching. Opt Express 19: 18861–18870. 21996828 10.1364/OE.19.018861

[mbt213359-bib-0018] Felsenstein, J. (1985) Confidence limits on phylogenies: an approach using the bootstrap. Evolution 39: 783–791. 28561359 10.1111/j.1558-5646.1985.tb00420.x

[mbt213359-bib-0019] Fu, H.Y. , Lin, Y.C. , Chang, Y.N. , Tseng, H. , Huang, C.C. , Liu, K.C. , *et al* (2010) A novel six‐rhodopsin system in a single archaeon. J Bacteriol 192: 5866–5873. 20802037 10.1128/JB.00642-10 PMC2976437

[mbt213359-bib-0020] Fu, H.Y. , Yi, H.P. , Lu, Y.H. , and Yang, C.S. (2013) Insight into a single halobacterium using a dual‐bacteriorhodopsin system with different functionally optimized pH ranges to cope with periplasmic pH changes associated with continuous light illumination. Mol Microbiol 88: 551–561. 23565724 10.1111/mmi.12208

[mbt213359-bib-0021] Grant, W.D. , Kamemkura, M. , McGenity, T.J. , and Ventosa, A. (2001) Class III halobacteria class, nov In Bergey's Manual of Systematic Bacteriology. Boone D.R., Castenholz R.W., and Garrity G.M. (eds). New York: Springer, pp. 294–334.

[mbt213359-bib-0022] Hong, F.T. (1994) Retinal proteins in photovoltaic devices In Molecular and Biomolecular Electronics. Birge R.R. (ed). Washington, DC: American Chemical Society, pp. 527–559.

[mbt213359-bib-0023] Hsu, M.F. , Yu, T.F. , Chou, C.C. , Fu, H.Y. , Yang, C.S. , and Wang, A.H. (2013) Using Haloarcula marismortui bacteriorhodopsin as a fusion tag for enhancing and visible expression of integral membrane proteins in Escherichia coli. PLoS ONE 8: e56363. 23457558 10.1371/journal.pone.0056363 PMC3574148

[mbt213359-bib-0024] Ikemura, T. (1981) Correlation between the abundance of *Escherichia coli* transfer RNAs and the occurrence of the respective codons in its protein genes: a proposal for a synonymous codon choice that is optimal for the *E. coli* translational system. J Mol Biol 151: 389–409. 6175758 10.1016/0022-2836(81)90003-6

[mbt213359-bib-0025] Kahaki, F.A. , Babaeipour, V. , Memari, H.R. , and Mofid, M.R. (2014) High overexpression and purification of optimized bacterio‐opsin from *Halobacterium Salinarum* R1 in *E. coli* . Appl Biochem Biotechnol 174: 1558–1571. 25123363 10.1007/s12010-014-1137-2

[mbt213359-bib-0026] Kanekar, P.P. , Kulkarni, S.O. , Kanekar, S.P. , Shouche, Y. , Jani, K. , and Sharma, A. (2015) Exploration of a haloarchaeon, *Halostagnicola larsenii*, isolated from rock pit sea water, West Coast of Maharashtra, India, for the production of bacteriorhodopsin. J Appl Microbiol 118: 1345–1356. 25727916 10.1111/jam.12784

[mbt213359-bib-0027] Karnik, S.S. , Nassal, M. , Doi, T. , Jay, E. , Sgaramella, V. , and Khorana, H.G. (1987) Structure‐function studies on bacteriorhodopsin. II. Improved expression of the bacterio‐opsin gene in *Escherichia coli* . J Biol Chem 262: 9255–9263. 3298253

[mbt213359-bib-0028] Karnik, S. , Doi, T. , Molday, R. , and Khorana, H.G. (1990) Expression of the archaebacterial bacterio‐opsin gene with and without signal sequences in *Escherichia coli*: the expressed proteins are located in the membrane but bind retinal poorly. Proc Natl Acad Sci USA 87: 8955–8959. 2247471 10.1073/pnas.87.22.8955 PMC55079

[mbt213359-bib-0029] Kelley, L.A. , Mezulis, S. , Yates, C.M. , Wass, M.N. , and Sternberg, M.J. (2015) The Phyre2 web portal for protein modeling, prediction and analysis. Nat Protoc 10: 845–858. 25950237 10.1038/nprot.2015.053 PMC5298202

[mbt213359-bib-0030] Kumar, S. , Stecher, G. , and Tamura, K. (2016) MEGA7: molecular evolutionary genetics analysis version 7.0 for bigger datasets. Mol Biol Evol 33: 1870–1874. 27004904 10.1093/molbev/msw054 PMC8210823

[mbt213359-bib-0031] Lanyi, J.K. , and Luecke, H. (2001) Bacteriorhodopsin. Curr Opin Struct Biol 11: 415–419. 11495732 10.1016/s0959-440x(00)00226-8

[mbt213359-bib-0032] Lindvold, L. , and Lausen, H. (2006) A projection display based on a bacteriorhodopsin thin film In Bionanotechnology: Proteins to Nanodevices. Renugopalakrishnan V., and Lewis R.V. (eds). Dordrecht: Springer, The Netherlands, pp. 79–95.

[mbt213359-bib-0033] Lozier, R.H. , Bogomolni, R.A. , and Stoeckenius, W. (1975) Bacteriorhodopsin: a light‐driven proton pump in *Halobacterium Halobium* . Biophys J 15: 955–962. 1182271 10.1016/S0006-3495(75)85875-9 PMC1334761

[mbt213359-bib-0034] Mandelli, F. , Miranda, V.S. , Rodrigues, E. , and Mercadante, A.Z. (2012) Identification of carotenoids with high antioxidant capacity produced by extremophile microorganisms. World J Microbiol Biotechnol 28: 1781–1790. 22805960 10.1007/s11274-011-0993-y

[mbt213359-bib-0035] Mao, D. , and Wallace, B.A. (1984) Differential light scattering and absorption flattening optical effects are minimal in the circular dichroism spectra of small unilamellar vesicles. Biochemistry 23: 2667–2673. 6466606 10.1021/bi00307a020

[mbt213359-bib-0036] Marque, J. , Eisenstein, L. , Gratton, E. , Sturtevant, J.M. , and Hardy, C.J. (1984) Thermodynamic properties of purple membrane. Biophys J 46: 567–572. 6498271 10.1016/S0006-3495(84)84055-2 PMC1435054

[mbt213359-bib-0037] Mogi, T. , Marti, T. , and Khorana, H.G. (1989) Structure‐function studies on bacteriorhodopsin. IX. Substitutions of tryptophan residues affect protein‐retinal interactions in bacteriorhodopsin. J Biol Chem 264: 14197–14201. 2547787

[mbt213359-bib-0038] Montalvo‐Rodriguez, R. , Vreeland, R.H. , Oren, A. , Kessel, M. , Betancourt, C. , and Lopez‐Garriga, J. (1998) *Halogeometricum borinquense* gen. nov., sp. nov., a novel halophilic archaeon from Puerto Rico. Int J Syst Bacteriol 48(Pt 4): 1305–1312. 9828431 10.1099/00207713-48-4-1305

[mbt213359-bib-0039] Nekrasova, O.V. , Wulfson, A.N. , Tikhonov, R.V. , Yakimov, S.A. , Simonova, T.N. , Tagvey, A.I. , *et al* (2010) A new hybrid protein for production of recombinant bacteriorhodopsin in *Escherichia coli* . J Biotechnol 147: 145–150. 20363267 10.1016/j.jbiotec.2010.03.019

[mbt213359-bib-0040] Oesterhelt, D. , and Stoeckenius, W. (1971) Rhodopsin‐like protein from the purple membrane of *Halobacterium halobium* . Nature New Biol 233: 149–152. 4940442 10.1038/newbio233149a0

[mbt213359-bib-0041] Oesterhelt, D. , and Stoeckenius, W. (1973) Functions of a new photoreceptor membrane. Proc Natl Acad Sci USA 70: 2853–2857. 4517939 10.1073/pnas.70.10.2853 PMC427124

[mbt213359-bib-0042] Opekarova, M. , and Tanner, W. (2003) Specific lipid requirements of membrane proteins–a putative bottleneck in heterologous expression. Biochem Biophys Acta 1610: 11–22. 12586375 10.1016/s0005-2736(02)00708-3

[mbt213359-bib-0043] Roosild, T.P. , Vega, M. , Castronovo, S. , and Choe, S. (2006) Characterization of the family of Mistic homologues. BMC Struct Biol 6: 10. 16704729 10.1186/1472-6807-6-10 PMC1471793

[mbt213359-bib-0044] Saitou, N. , and Nei, M. (1987) The neighbor‐joining method: a new method for reconstructing phylogenetic trees. Mol Biol Evol 4: 406–425. 3447015 10.1093/oxfordjournals.molbev.a040454

[mbt213359-bib-0045] Stuart, J.A. , Tallent, J.R. , Tan, E.H.L. and Birge, R.R. (1996) Protein‐based volumetric memories In: Proceedings of Nonvolatile Memory Technology Conference, pp. 45–51.

[mbt213359-bib-0046] Stuart, J.A. , Marcy, D.L. , Wise, K.J. , and Birge, R.R. (2002) Volumetric optical memory based on bacteriorhodopsin. Synth Met 127: 3–15.

[mbt213359-bib-0047] Thombre, R.S. , Shinde, V.D. , Oke, R.S. , Dhar, S.K. , and Shouche, Y.S. (2016) Biology and survival of extremely halophilic archaeon Haloarcula marismortui RR12 isolated from Mumbai salterns, India in response to salinity stress. Sci Rep 6: 25642. 27231230 10.1038/srep25642 PMC4882750

[mbt213359-bib-0048] Wang, W.W. , Sineshchekov, O.A. , Spudich, E.N. , and Spudich, J.L. (2003) Spectroscopic and photochemical characterization of a deep ocean proteorhodopsin. J Biol Chem 278: 33985–33991. 12821661 10.1074/jbc.M305716200

[mbt213359-bib-0049] Xu, J. , Stickrath, A.B. , Bhattacharya, P. , Nees, J. , Varo, G. , Hillebrecht, J.R. , *et al* (2003) Direct measurement of the photoelectric response time of bacteriorhodopsin via electro‐optic sampling. Biophys J 85: 1128–1134. 12885657 10.1016/S0006-3495(03)74549-4 PMC1303231

