# Supplementary material for: Discovery of bacteriorhodopsins in Haloarchaeal species isolated from Indian solar salterns: deciphering the role of the N‐terminal residues in protein folding and functional expression
Source: Microb Biotechnol. 2019 Jan 16;12(3):434–46. doi: 10.1111/1751-7915.13359 (PMC6465532; doi:10.1111/1751-7915.13359)
Supplement: Supplementary file 1 — Fig. S1. Morphological variations observed in the haloarchaeal isolates. Fig. S2. Schematic presentation of the Mistic‐BR fusion constructs designed and used in the study. Fig. S3. In‐gel digestion and ms/ms ion search for K1T BR. Fig. S4. Heat denaturation of the wild‐type H. salinarum BR and K1T bop. Fig. S5. Operon analysis of bop. Fig. S6. Operon schematics of V‐type ATPases in (A) H. borinquense (B) H. marismortui (C) H. rufum, showing the bop position in between the B and D subunits of the V‐type ATPases. Table S1. Forward and reverse primers for 16S rRNA amplification, degenerative primers, full‐length bop amplification and gene grafting. [file MBT2-12-434-s001.docx]

**Discovery of bacteriorhodopsins in Haloarchaeal species isolated from Indian solar salterns: deciphering the role of the N-terminal residues in protein folding and functional expression**

Dipesh Kumar Verma^1^, Ishita Baral^2^, Atul Kumar^2^, Senthil E. Prasad^2#^ and Krishan Gopal Thakur^1#^

^1^G. N. Ramachandran Protein Centre, Structural Biology Laboratory, Council of Scientific and Industrial Research-Institute of Microbial Technology (CSIR-IMTECH), Chandigarh-160036, India

^2^Biochemical Engineering Research and Process Development Centre, Council of Scientific and Industrial Research-Institute of Microbial Technology (CSIR-IMTECH), Chandigarh-160036, India

**Correspondence**

**^#^Krishan Gopal Thakur**

[Email: krishang@imtech.res.in](mailto:Email:%20krishang@imtech.res.in)

Phone: +91-172-6665470

^#^Senthil E. Prasad

[Email:esprasad@imtech.res.in](mailto:#esprasad@imtech.res.in)

Phone: +91-172-6665223

**Running title: Recombinant expression of haloarcheal bacteriorhodopsins**

**Supplementary Figures
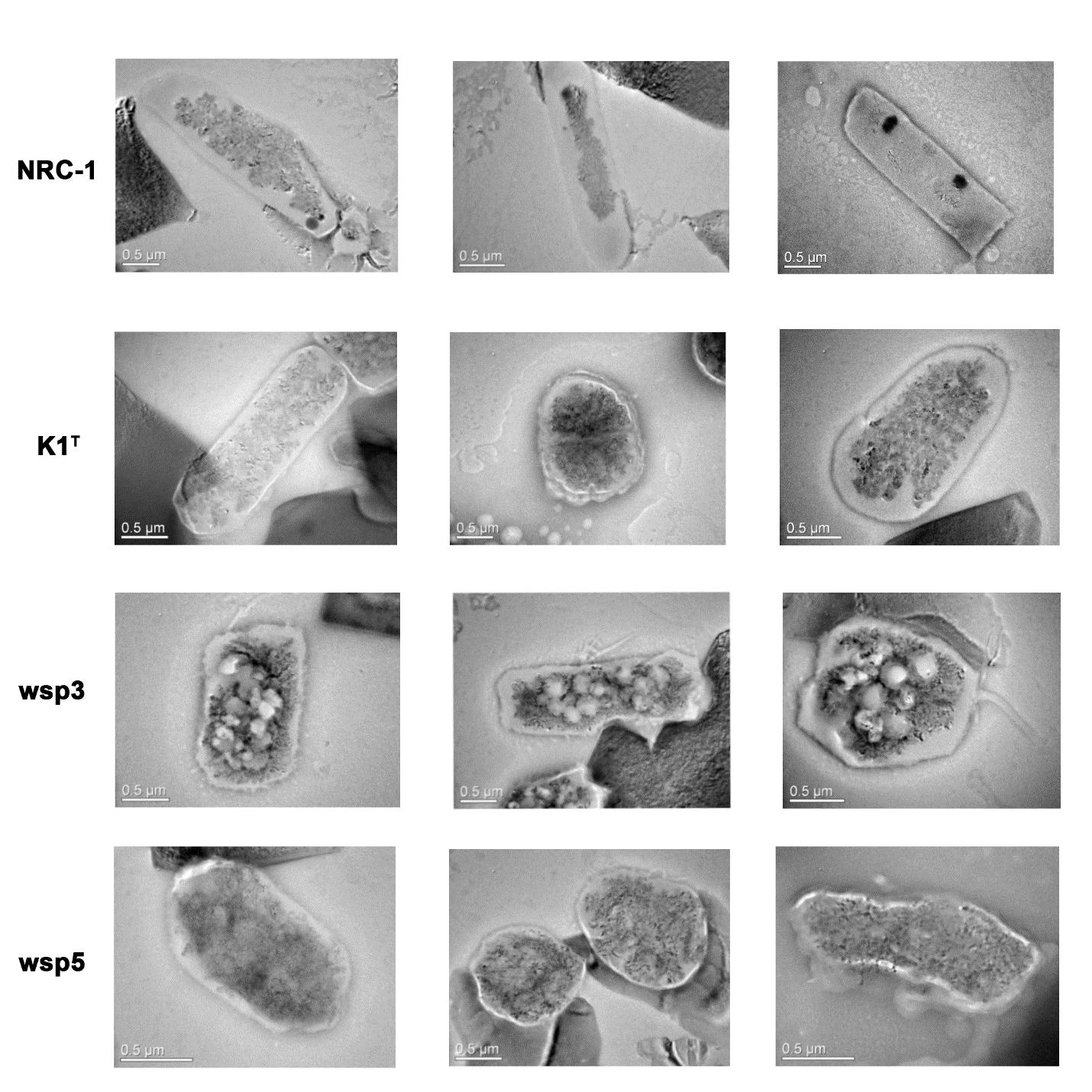
**

**Figure S1.** Morphological variations observed in the haloarchaeal isolates. The *Halogeometricum* sp. wsp3 isolate appeared to be highly vacuolated compared with the other isolates.

**
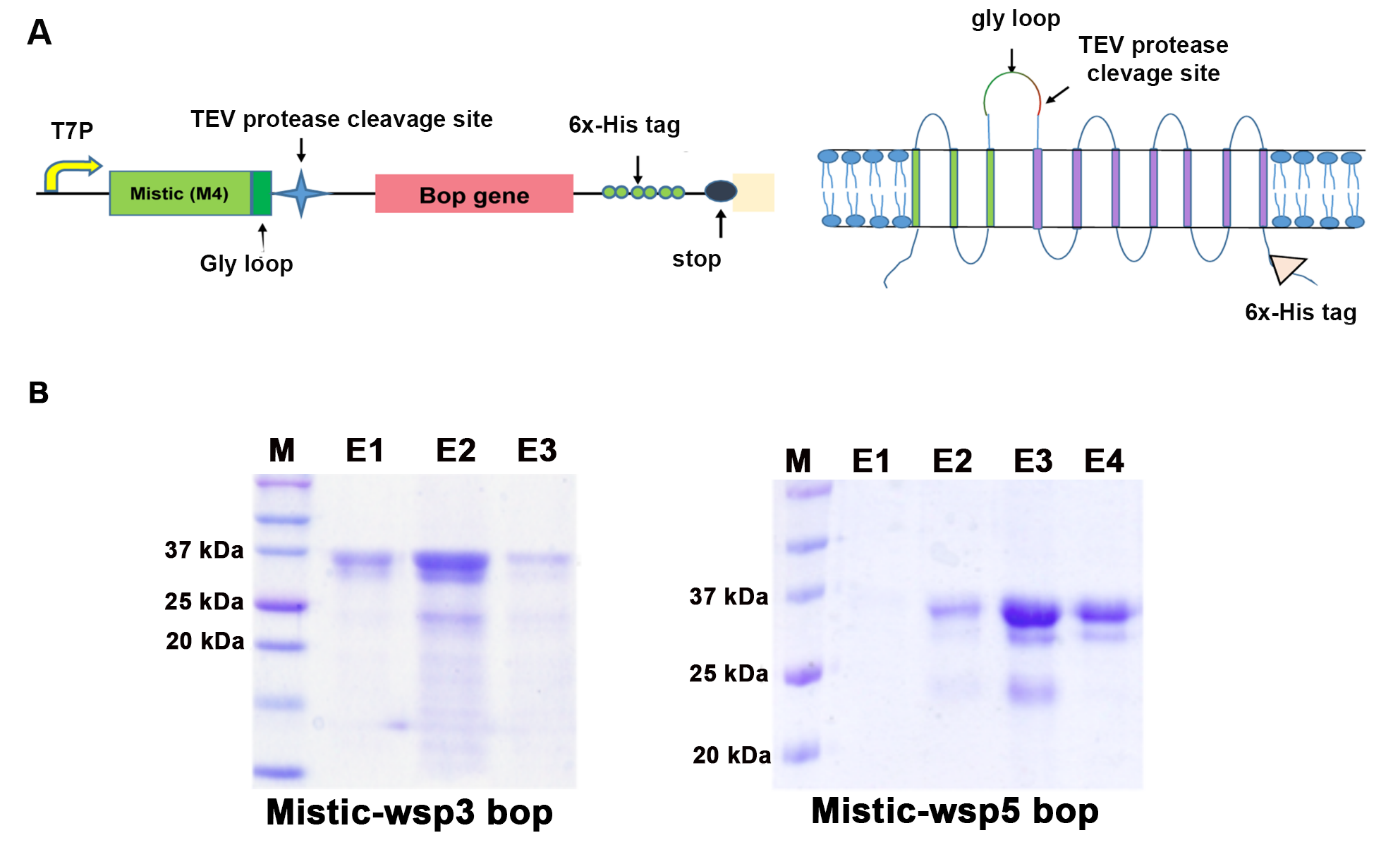
**

**Figure S2. Schematic presentation of the Mistic-BR fusion constructs designed and used in the study. A)** The pET28a vector was modified by adding the Mistic gene (M4) as an N-terminal fusion tag. The tag was separated from BR using the TEV protease cleavage site. **B)** The purified M4-BR fusion proteins showed bands as expected at ~37 kDa on SDS-PAGE. E1, E2, E3 and E4 are step elution fractions of M4-BR proteins, eluted with increasing concentration (20 mM, 100 mM, 200 mM and 500 mM, respectively) of imidazole. M lane corresponds to the standard molecular weight marker.

**
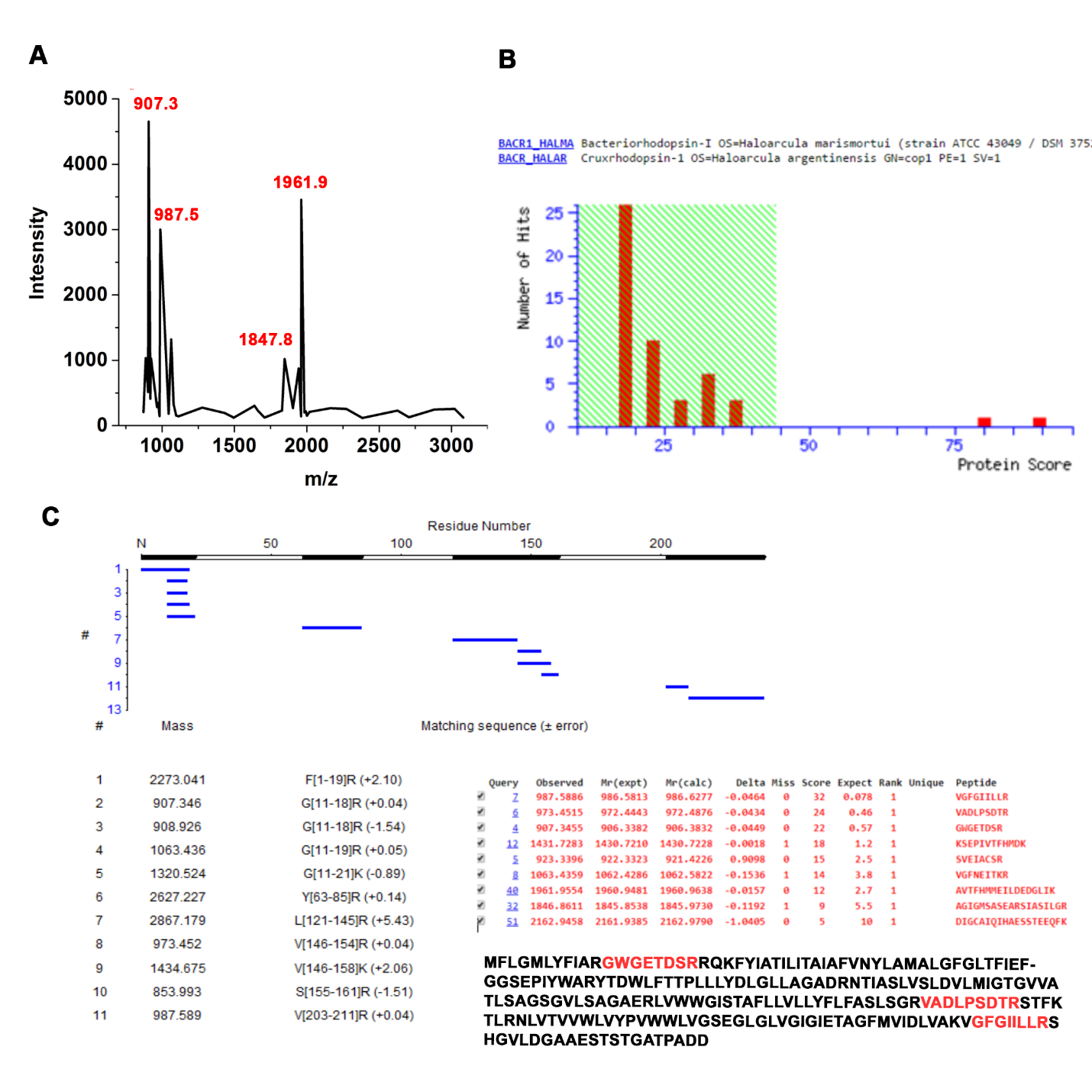
**

**Figure S3. In-gel digestion and ms/ms ion search for K1^T^ BR: A) & B)** The intensity peaks and Mascot protein database search results suggested that the purified protein was BR. **C) & D)** The masses were further confirmed using the PAWS server and showed a possible fragment match with BR.

**
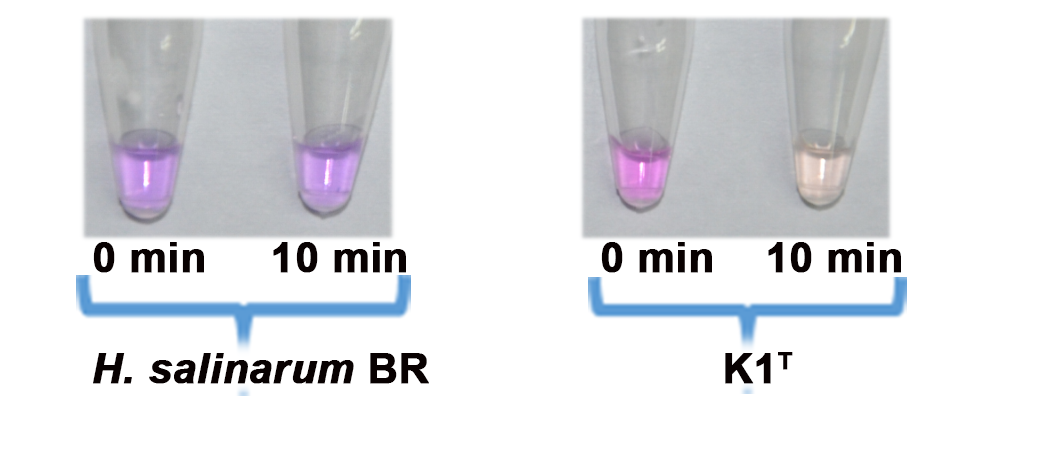
**

**Figure S4**. Heat denaturation of the wild-type *H. salinarum* BR and K1^T^ bop. Both BRs were heated at 75 ºC for 10 min. K1^T^ BR shows a colour loss, while *H. salinarum* BR is stable.


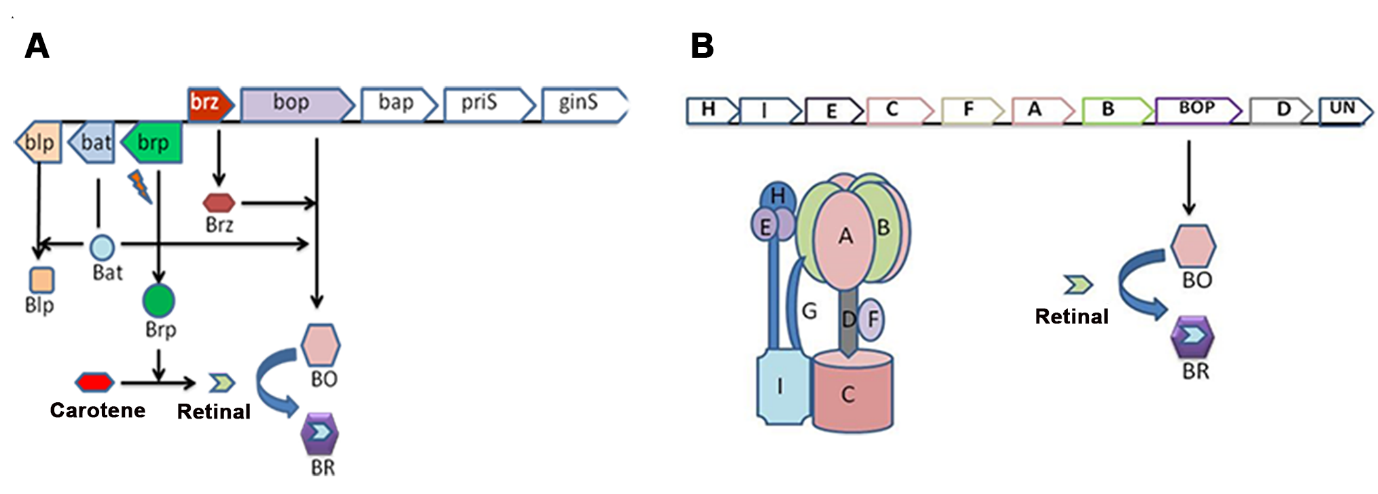


**Figure S5.** Operon analysis of *bop*. **A)** *Halobacterium salinarum* R1 *bop* operon showing *blp* (Bacterio-opsin-linked protein), *bat* (BR transcriptional activator) and *brp* (beta-carotene 15, 15’-dioxygenase) accessory genes. **B)** Representation of *Haloarcula* sp*.* K1^T^ showing *bop* in between the B and D subunits of a V-type ATPase.


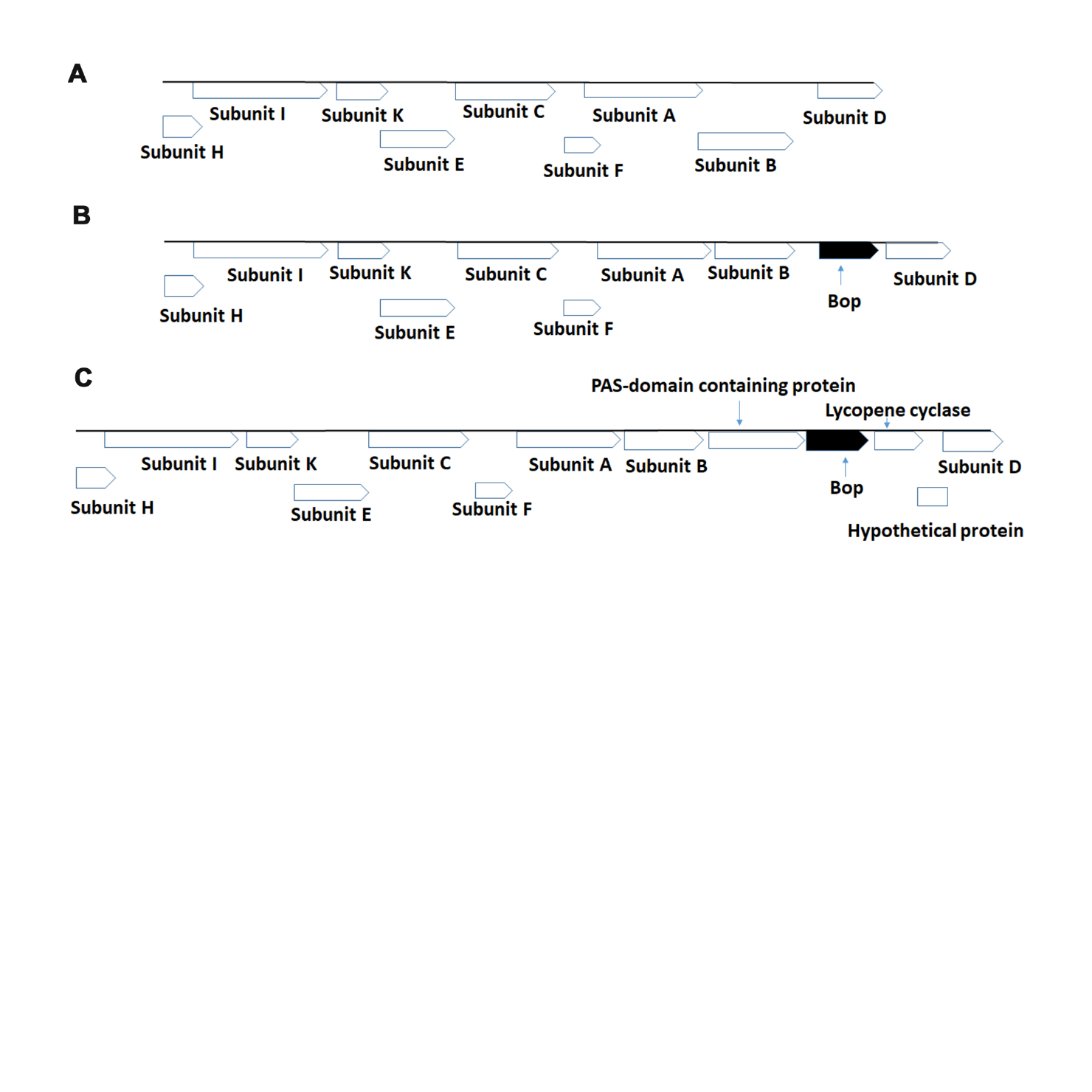


**Figure S6**. Operon schematics of V-type ATPases in **A)** *H. borinquense* **B)** *H. marismortui* **C)** *H. rufum,* showing the *bop* position in between the B and D subunits of the V-type ATPases. The absence of *bop* in *H. borinquense* confirms its random insertion in the *H. marismortui* genome**.**

**Tables**

**Table S1.** Forward and reverse primers for 16S rRNA amplification, degenerative primers, full-length *bop* amplification and gene grafting.

| **Name** | **Primer sequence** |
| --- | --- |
| **21Fw** | TCCGGTTGATCCYGCCGG |
| **1453Rev** | GGGCYGCACGCGYRCTACA |
| **DegFw** | GACTGGTTGTTYATVACGCC |
| **DegRev** | AASCCGAAGCCGAYCTTBGC |
| **Bop_full_Fw** | ACCGAAGCTAGCTTCCTCGGCATGCTCTACTTCATC |
| **Bop_full_Rev** | CTTGCTCGAGGTCGTCTGC AGGCGTTG CGCC |
| **1^st^ variant** | GTATCGCGCCCAGTAGATCGGATGCTGTTCACCACC |
| **2^nd^ variant** | GGTTGGGGTGAAACCGATAGCAGGCGTCAGAAGTTC |
| **3^rd^ variant** | TGGGCACCGCAGGTATGTTCCTCGGCATGCTCTACTT |
